# Developing and validating a multidimensional Chinese Parental Psychological Control Scale

**DOI:** 10.3389/fpsyg.2023.1116625

**Published:** 2023-03-22

**Authors:** Xiaoqin Zhu, Diya Dou, Yangu Pan

**Affiliations:** ^1^Department of Applied Social Sciences, The Hong Kong Polytechnic University, Hong Kong, Hong Kong SAR, China; ^2^Research Institute of Social Development, Southwestern University of Finance and Economics, Chengdu, China

**Keywords:** psychological control, Chinese contexts, scale validation, multidimensions, invariance test

## Abstract

**Introduction:**

This study validated a Chinese Parental Psychological Control Scale (CPPCS) among secondary school students in mainland China.

**Methods:**

The item pool consisting of 65 items was constructed based on consultation with existing measures and focus group interviews of 19 Chinese adolescents. After content validation conducted by 14 experts, a total of 40 items were retained and subject to further factorial validation based on a sample of 963 Chinese adolescents (mean age = 13.39 ± 0.72; 52.23% females).

**Results:**

Using the two random-split half samples, exploratory and confirmatory factor analyses retained 30 items that loaded on three factors, including “relational induction” (twelve items), “harsh psychological control” (twelve items), and “social comparison shame” (six items). The three-factor structure was invariant across gender (male versus female) and grades (grade 7 versus grade 8) among the whole sample. Cronbach’s αs of the three dimensions in maternal and paternal subscales ranged between 0.89 and 0.92, suggesting adequate internal consistency. The three dimensions were significantly correlated with each other, supporting the scale’s convergent validity. The concurrent validity of the CPPCS was supported by the positive correlations between subscales and parental rejection, and the negative correlations between subscales and parental warmth. In addition, while the “harsh psychological control” and “social comparison shame” negatively predicted adolescents’ well-being, “relational induction” positively predicted adolescents’ well-being.

**Discussion:**

The findings suggest that the CPPCS is a promising instrument for measuring multidimensional psychological control among Chinese parents and for investigating and comparing individual dimensions’ effect on adolescents’ development.

## 1. Introduction

Unfavorable adolescent developmental outcomes, including poor well-being (e.g., low life satisfaction) and growing ill-being (e.g., depression), particularly exacerbated by COVID-19, have become a worrying social issue in Chinese and other societies. Understanding what factors may contribute to or act against this trend and in what ways is of great concern to parents, scholars, youth workers, and policymakers. As parents are the primary socialization agents, parenting remains one of the focuses of inquiry.

Among different parenting strategies, parental psychological control has been the subject of heated discussion. In contrast to parental behavioral control which exerts due parental authority and regulations over children’s activities and behaviors and is often associated with favorable child developmental outcomes, psychological control refers to a type of dysfunctional parental control that represents parental undue authority over children’s thoughts and feelings through multiple intrusive and manipulative tactics (Barber and Harmon, 2002; Barber et al., 2005). These tactics include “invalidating children’s feelings” (discounting children’s feelings and thoughts), “constraining verbal expression” (preventing or interrupting children’s expression), “personal attack” (attacking children’s self-worth and identity), shaming and guilty induction (evoking children’s feelings of guilty or ashamed), and love withdrawal (threatening the loss or actual loss of parental love or attention) (Barber and Harmon, 2002; Barber et al., 2005). Characterized by these tactics, psychological control has been conceptualized as manipulation and coercion, intrusion into the personal domain, and disrespect of individuality (Barber et al., 2012).

Parental psychological control negatively affects children’s development because it violates their psychological world and sense of self; it also makes children feel pressured, inferior, and alienated, thwarting their basic psychological needs (Soenens and Vansteenkiste, 2010). In Western samples, parental psychological control is generally associated with the child’s unfavorable developmental outcomes indicated by low well-being and high ill-being (see Scharf and Goldner, 2018 for a review). Yet, the findings in Chinese settings are equivocal despite that parental psychological control, especially in shame and guilt induction forms, is commonly practiced by Chinese parents. While some studies reported similar negative impacts of parental psychological control (Shek, 2006; Yao et al., 2022), some others failed to do so (Shek and Zhu, 2019; Zhu and Shek, 2020). In some cross-cultural studies, psychological control hindered children’s healthy functioning across cultures including in China (Barber et al., 2005; Wang et al., 2007). Other cross-cultural studies identified negative impacts of parental psychological control only in Western contexts, but not in Asian ones (Olsen et al., 2002; Rudy and Halgunseth, 2005). A recent meta-analysis claimed that culture (Western vs. Eastern) did not moderate the effects of psychological control on child problem behaviors (Yan et al., 2020), but this is based on only two Chinese studies.

One possible explanation for the above-mentioned mixed findings is that some forms of parental psychological control may be universally harmful, while others may function differently in Chinese contexts. While Western cultures value independence, autonomy, and individuality as essential building blocks of self-construal and healthy child development, Chinese culture generally prioritizes harmonious inter-relationships and interdependence in the family. It is noted that Chinese parents tend to feel obligated to manage their children’s lives, such as ensuring their academic excellence, social, emotional, and behavioral adjustment, and helping them “fit in” and be a part of society (Cheung and Pomerantz, 2011). As such, some forms (e.g., guilt induction) may be practiced to realize prevailing socialization priorities in Chinese contexts, which have been argued to be well-intended rather than out of rejection and hostility, and thus less detrimental (Scharf and Goldner, 2018; Ng and Wang, 2019). For example, the feeling of guilt is regarded as one important element in morality and an indicator of filial piety in Chinese cultures, which helps achieve interpersonal obligations (Chen et al., 2016). However, most existing Chinese studies failed to capture the various dimensions of parental psychological control and explore their distinctive effects.

First, the investigations of parental psychological control in most Chinese studies were based on Western frameworks, with many (e.g., Barber et al., 2005; Shek, 2006; Yu et al., 2021) assessing psychological control through a translated or updated “Psychological Control Scale” (PCS; Barber, 1996), which included eight items on *constraining verbal expression*, *invalidation*, *love withdrawal*, and *personal attack*. Such investigations may be appropriate in Western contexts, but they exclude shaming and guilt induction, which are commonly used by Chinese parents. Hence, parental psychological control has not been defined and measured to its conceptual fullness. Second, despite an initial emphasis on its multifaceted nature, parental psychological control has often been empirically reduced to a unidimensional structure because of the narrow scope and the limited number of items of global measures. Although some studies assessed psychological control multidimensionally (Wang et al., 2007; Yao et al., 2022), they selected only three to four relevant forms while excluding others and treated each form as an individual dimension without exploring the co-grouping of different forms. For example, the frequently used scale developed by Wang et al. (2007) tapped three dimensions: guilt induction, love withdrawal, and authority assertion. Furthermore, the selected dimensions were usually used to construct a global parental psychological control index, assuming similar functions for all dimensions. This may be problematic, as different dimensions may have distinct effects. For example, guilt induction was contrasted with love withdrawal regarding its implications among Chinese adolescents (Yu et al., 2019).

Based on the above, a well-grounded and validated multidimensional construction of parental psychological control is essential for resolving the current controversy and identifying its detrimental and more permissible dimensions in Chinese contexts. Fung and Lau (2012) differentiated “harsh psychological control” from “relational induction” in Chinese contexts. The former (e.g., constraining verbal expression, invalidation, and personal attack) denotes parental hostility and rejection toward children and is likely to be universally detrimental. The latter (e.g., guilt induction, shaming, and love withdrawal) may be less harmful in Chinese settings since it reflects parental attempts to ensure that children meet societal norms by enhancing their understanding of what others think of them. In line with this theoretical expectation, harsh psychological control was more strongly linked to parental rejection than relational induction among Chinese parents (Fung and Lau, 2012).

Fung and Lau’s (2012) framework provided a conceptual basis for understanding the unique dimensionality of parental psychological control in Chinese contexts. However, the authors did not empirically explore how different forms of psychological control are grouped into the two dimensions. Instead, they preassigned selected items to each dimension, which is threefold problematic. First, it may not have captured the conceptual fullness of the dimensions, resulting in unexpectedly insignificant effects of harsh psychological control. Specifically, harsh psychological control was defined mainly based on the “Psychological Control Scale” mentioned earlier but excluded other hostile forms, such as disregarding or depreciating children’s thoughts (e.g., “the child’s thoughts are naive”), which Chinese adolescents perceived as dismissive (Sze, 2016).

Second, love withdrawal was grouped with shaming and guilt induction under “relational induction,” since they might facilitate children’s reflection on and correction of misbehavior (Fung and Lau, 2012). This pre-assignment is open to question. Shaming and guilt induction are thought to be socialization strategies employed by Chinese parents to instill social sensitivity and responsibility in children (Fung, 1999). Love withdrawal is theoretically more aversive and harmful because it centers on the threat of the loss of parental love, which reflects conditional parental acceptance and potential rejection (Yu et al., 2015). Empirically, while guilt and shame induction did not show negative impacts (Fang et al., 2022) and even exerted positive influences (Yu et al., 2019), love withdrawal consistently showed negative effects on Chinese adolescents’ development (Cheah et al., 2019; Yu et al., 2019). Thus, love withdrawal is arguably different from relational induction (Fang et al., 2022).

Third, there is a need to distinguish between “shared shame” and “social comparison shame.” While the former (e.g., “the child’s behavior makes parents lose face”) focuses on the influence of children’s misdeeds on parental or familial reputation, the latter (e.g., “the child is not as good as another kid”) reflects parental disappointment by comparing the child unfavorably to others. Shared shame may be more benign in Chinese contexts as it is used to foster children’s identification with salient moral and social norms, such as reciprocity and interdependent familial relations (Yu et al., 2019). In contrast, social comparison shame, which compares children’s shortcomings to those of others, may imply parental disrespect and rejection (Smetana et al., 2021). It is likely to convey essentially the same core message as harsh psychological control (i.e., the child is not good), thus hurting the child similarly.

Based on the above elaborations, there is a need to develop and validate an indigenous Chinese multidimensional parental psychological control scale that sufficiently covers all essential forms of parents’ psychological control. Thus, this study aimed to first construct an instrument to measure parental psychological control to its conceptual fullness and then validate the scale and examine its psychometric properties among Chinese adolescents. We expected adequate reliability (e.g., internal consistency) of subscales taping different dimensions of parental psychological control, which indicates that the included items measure a homogenous construct. For validity, we first examined the factorial validity through both exploratory and confirmatory factor analyses (EFA and CFA) as well as invariances tests to confirm the grouping of items under different dimensions. In addition, we examined the new instrument’s convergent validity by checking the correlations among individual dimensions and its concurrent validity as the correlations between dimensions and other parental factors (parental rejection and warmth). We also performed a preliminary investigation of differentiated prediction effects of individual dimensions of parental psychological control on adolescents’ developmental outcomes. Based on the aforementioned elaborations, individual dimensions were expected to be positively correlated with each other, positively correlated with parental rejection, and negatively correlated with parental warmth. In addition, some dimensions (e.g., those in hostile forms) would show stronger negative predictions on adolescents’ developmental outcomes than relatively well-intended dimensions (e.g., those related to shared shame).

## 2. Methods

### 2.1. Construction of item pool

To construct an item pool with full coverage of essential forms of parental psychological control, the research team consulted the existing measures on parental psychological control (e.g., Shek, 2006; Wang et al., 2007; Sze, 2016; Yu et al., 2019; Zhu and Shek, 2020; Fang et al., 2022) and Chinese adolescents’ experiences. First, a list of 60 Chinese items pertinent to domineering control, invalidation, ignoring, personal attack, constraining verbal expression, guilt induction, shared shame, love withdrawal, and social comparison shame were derived from prior studies. Second, based on the recommendations from Mallinckrodt et al. (2016) on improving item quality in scale development, the research team conducted three focus group interviews involving 19 Chinese secondary school students (10 females, mean age = 12.05, SD = 1.35). Barber et al. (2012) remarked that “one of the most fundamental measurement limitations of the construct of psychological control to date; namely that youth – the recipients of the control – have not systematically been consulted when defining items to be used to measure it” (p. 276). As adolescents are the ones who experience and are influenced by parental psychological control, taking into account how they define and perceive parental psychological control can help get more informative and accurate items.

During the focus group interviews, the first author explained definitions of parental psychological control and its different forms and presented the list of the 60 items in Chinese to the participants, who subsequently, shared their interpretations of the items and understandings of different manifestations of parental psychological control they had experienced or observed. The participants’ responses were carefully reviewed by the research team and used to enrich the item pool and modify certain wordings for easy and accurate comprehension among adolescent participants. As a result, ten items were modified for better understanding and five additional items were created for a more complete pool. Thus, the final item pool of 65 items was subject to further content validation.

Specifically, 14 researchers in Psychology or Education evaluated each item regarding their representativeness, relevance, and clarity and provided suggestions on item modification if deemed necessary. Items rated as unrepresentative, irrelevant, or unclear by any researcher were subject to further review and refinement by the research team. Consequently, five items were revised for better clarity, five items were discarded as they were not sufficiently relevant to or representative of parental psychological control, and another twenty items were also removed because their meanings were repetitive or similar to other items. The retained and revised 40 items were distributed to the researchers again and all the researchers rated that the items were clear, relevant, and representative. Thus, these 40 items formed the Chinese Parental Psychological Control Scale (CPPCS), which was subject to further factorial validation. Among the 40 items, 35 were derived from prior studies and five were newly produced in this study. All items were translated into English following standard translation and back-translation procedures (Brislin, 1980).

### 2.2. Participants and procedures

To further validate the 40-item CPPCS and investigate its psychometric properties, 963 adolescents in grades 7 and 8 (junior secondary one and two) were recruited from four secondary schools in four cities, respectively, in Mainland China. The four participating junior secondary schools were invited using a convenience sampling strategy. Then, the responsible teacher in each school further invited a few classes in grades 7 and 8 to participate in the study (grades 9 students were not invited because they were busy engaged in preparing for high school entrance examination). The mean age of the participants was 13.39 (SD = 0.72). Among the participants, 460 (47.77%) were male adolescents and the other 503 (52.23%) were female adolescents. The participants were invited to complete a questionnaire in their classrooms, including CPPCS and other measures in a paper-and-pencil format. The schools, parents, and adolescents provided written consent for adolescents’ participation. Ethical approval (HSEARS20220427002) was obtained from Institutional Review Board in the first author’s university.

### 2.3. Measures

The questionnaire included multiple measures, covering CPPCS and additional measures. For the additional measures, parental warmth and rejection were measured to investigate the concurrent validity of the CPPCS. Three indicators of child developmental outcomes (self-esteem, life satisfaction, and depression) were also measured to primarily test potential differentiated predictions of individual dimensions in the CPPCS.

*Chinese Parental Psychological Control Scale (CPPCS)* employed a five-point Likert Scale (“1 = never”; “5 = always”) for the 40 items to assess parents’ psychological control perceived by adolescents. The participants rated maternal and paternal psychological control separately.

*Parental Warmth* and *Parental Rejection* were measured by the Chinese short form of the Egna Minnen Beträffande Uppfostran (s-EMBU), which has been validated and widely used among Chinese children and adolescents (Guo et al., 2021). Subscales of warmth (6 items, e.g., “My parents praised me”) and rejection (7 items, e.g., “My parents were sour or angry with me without letting me know the cause”) were utilized in this study. Paternal and maternal warmth as well as paternal and maternal rejection were assessed separately. A five-point rating scale (1 = “strongly disagree” to 5 = “strongly agree”) was used. CFA showed that the two-factor structure of parental warmth and rejection fitted the data adequately in the current study (maternal: *χ^2^_(64)_* = 163.629, CFI = 0.98, TLI = 0.97, RMSEA = 0.04, SRMR = 0.04; paternal: *χ^2^_(64)_* = 164.52, CFI = 0.97, TLI = 0.97, RMSEA = 0.04, SRMR = 0.04). The internal consistency of the warmth subscales (*α*_father_ = 0.85/*ω*_father_ = 0.86; *α*_mother_ = 0.86/*ω*_mother_ = 0.86) and rejection subscales (*α*_father_ = 0.83/*ω*_father_ = 0.83; *α*_mother/_ = 0.82/*ω*_mother_ = 0.83) was adequate in this study.

*Self-esteem* was measured by the Chinese version of the Rosenberg Self-Esteem Scale (RSES). The original RSES consisted of 10 items with five reverse keyed. Previous studies among Chinese samples suggested the adoption of the five positively worded items (e.g., “I am able to do things as well as most other kids” and “I feel that I’m as good as other kids”) for better reliability (e.g., Sze, 2016). Following the recommendation, the present study used the five positively worded items. The participants indicated the extent to which they agreed with each statement on a four-point scale (1 = “strongly disagree” to 4 = “strongly agree”). A higher mean score across the items indicated greater global self-esteem. In the present study, the one-factor structure of the five items fitted the data adequately: *χ^2^_(5)_* = 7.86, CFI = 0.99, TLI = 0.99, RMSEA = 0.03, SRMR = 0.01. The Cronbach’s alpha and McDonald’s omega values for the self-esteem scale were both 0.88 in the present study.

*Life Satisfaction* was measured by the 5-item Chinese “Satisfaction with Life Scale” in terms of participants’ subjective appraisal of their overall quality of life (e.g., “The conditions of my life are excellent” and “I am satisfied with my life”). The scale has been widely adopted in prior studies involving Chinese adolescents (e.g., Zhu and Shek, 2020; Zhou et al., 2021). A 6-point rating scale was used (1 = “strongly disagree” to 6 = “strongly agree”). In the present study, the one-factor structure of the scale fitted the data adequately: *χ^2^*_(5)_ = 16.30, CFI = 0.99, TLI = 0.98, RMSEA = 0.06, SRMR = 0.02. The Cronbach’s alpha and McDonald’s omega values of the scale in this study were both 0.80.

*Depression* was assessed by the Chinese version of the 10-item “Center for Epidemiological Studies-Depression” scale (CESD-10), a simplified form of the original 20-item CES-D. The CESD-10 has shown adequate reliability and validity for Chinese adolescents (e.g., Wang et al., 2021). The respondents indicated the frequency they displayed for each symptom described in the 10 items, including two reverse-keyed items, during the past week on a four-point scale (0 = “rarely or less than 1 day” to 3 = “most or all of the time or 5–7 days”). A higher total score across the items indicated a higher level of depression. In the present study, the CESD-10 showed a one-factor structure that fitted data adequately: *χ^2^*_(35)_ = 165.31, CFI = 0.95, TLI = 0.94, RMSEA = 0.07, SRMR = 0.03. The scale also demonstrated adequate internal consistency (*α* and *ω* = 0.83).

### 2.4. Data analysis

As we used a five-point reporting scale for CPPCS, the assumption of continuity for using maximum likelihood (ML) estimation can be met (Flora and Curran, 2004; Lubke and Muthén, 2004). Before examining the factorial validity of the CPPCS, we checked the skewness and kurtosis of participants’ responses on all 40 items. The absolute values of skewness (0.19–1.94) and kurtosis (0.01–4.24) met the requirements of normality (i.e., below 2 and 7, respectively). As a result, ML estimation can be correctly used in EFA and CFA (Finney and DiStefano, 2006). While EFA was performed using SPSS Version 26.0 (IBM Corp., Somers, NY, USA), CFA was performed using Mplus 8.5. As only 16–28 (1.66–2.91%) of the participants had variable-level missing values in CPPCS, the missing data were handled by mean imputation in EFA and “full information maximum likelihood estimation” incorporated in Mplus which enables the full usage of all available data in analyses (Cham et al., 2017).

First, EFA was performed based on the first random-split half sample (i.e., subsample A, *n* = 481) to explore factor structure and remove problematic items having serious double loadings or having factor loadings below 0.40 (Costello and Osborne, 2005). As different dimensions of psychological control are expected to be correlated with each other, we utilized Principal Axis Factoring with Promax Rotation.

Second, CFA was performed based on the second half sample (i.e., subsample B, *n* = 482) to further test the factor structure resultant from EFA in comparison to a unidimensional model where all retained items were loaded on a single factor. Following previous practices in scale validation research (Shek and Ma, 2010; Zhu et al., 2021), the difference in “Bayesian information criterion” (BIC) was used for deciding which model fitted the data better with 10 points smaller in BIC indicating 150:1 likelihood (*p* < 0.05) of preference (Schermelleh-Engel et al., 2003). Indices and criteria reflecting adequate model fit adopted in the present study included “Comparative Fit Index (CFI),” “Tucker-Lewis Index (TLI),” “Root Mean Square Error of Approximation (RMSEA),” and “Standardized Root Mean Square Residual (SRMR)” (CFI and TLI ≥ 0.90; RMSEA and SRMR ≤0.08).

Third, using the full sample (*N* = 963), invariance across gender and grade, respectively, for the confirmed factor structure was tested sequentially, including configural (free estimation), metric (equality constraints on factor loadings), and scalar (additional equality constraints on item intercepts) invariances (Svetina et al., 2020). As recommended by literature (Cheung and Rensvold, 2002; Meade et al., 2008), differences in CFI and RMSEA between two nested models and the related criteria (i.e., ∆CFI < 0.01 and ∆RMSEA <0.015) were used to determine factorial invariance. Inter-correlations among the factors were investigated to examine the scale’s convergent validity. A good convergent validity was also indicated by “average variance extracted” (AVE) ≥ 0.50, which means that at least 50% of the variance in the observed items is explained by the latent factors rather than residuals (Fornell and Larcker, 1981). Concurrent validity was also examined by checking correlations between the latent psychological control dimensions and the other two parental factors. Indicators of scale reliability included “composite reliability” (CR ≥ 0.70, Fornell and Larcker, 1981), Cronbach’s α, McDonald’s ω, and mean inter-item correlations. Finally, structural equation modeling was used to separately test the predictions of maternal and paternal psychological control dimensions on the three indicators of adolescents’ developmental outcomes.

## 3. Results

### 3.1. EFA

The KMO values (0.959 and 0.963 for maternal and paternal subscales, respectively) and Bartlett’s Test of Sphericity (*p*s < 0.001) suggested that the scale was highly factorable. The results of EFA and scree plot for the paternal subscale supported the extraction of three factors with initial eigenvalues above 1.0. For both subscales, the same ten items meeting the exclusion criteria mentioned earlier (seriously double-loaded or factor loadings <0.40) were removed, resulting in 30 items in the refined scale. Results after rotation are shown in Table 1.

**Table 1:** Factor loadings for retained items in the CPPCS after Promax Rotation (subsample A; *n* = 481).

Items	Maternal				Paternal			
	RI	HPC	SCS	Com.	RI	HPC	SCS	Com.
1. When I do not do things their way, my parents say that I make them unhappy	**0.74**	0.09	−0.09	0.56	**0.72**	0.13	−0.09	0.56
2. My parents expect me to be grateful and that I should not disappoint them	**0.73**	−0.05	0.06	0.55	**0.75**	−0.05	0.09	0.60
3. When I do not meet my parents’ expectations, they say that their sacrifice does not worthy	**0.72**	−0.03	0.02	0.51	**0.69**	0.04	−0.04	0.52
4. My parents say that if I really care for them, I would not do things that cause them to worry	**0.70**	−0.11	−0.08	0.35	**0.66**	−0.07	−0.06	0.36
5. My parents tell me all the things they have done for me	**0.66**	−0.11	0.17	0.62	**0.56**	−0.09	0.25	0.61
6. My parents tell me that they sacrifice much for me	**0.64**	−0.07	0.12	0.61	**0.56**	−0.03	0.19	0.59
7. My parents tell me that they get embarrassed in front of others when I do not meet their expectations	**0.56**	0.16	−0.02	0.52	**0.55**	0.18	0.00	0.49
8. My parents say that they will love me more if I perform better	**0.54**	0.05	0.00	0.40	**0.69**	−0.03	−0.05	0.42
9. My parents tell me I have to do well to honor the family	**0.52**	0.01	0.15	0.46	**0.57**	−0.03	0.18	0.53
10. My parents tell me that what they want me to do is the best for me, so I need to follow their demands	**0.42**	0.01	0.19	0.40	**0.44**	−0.08	0.27	0.39
11. My parents tell me that if I misbehave, people will think they are not good parents	**0.41**	0.01	0.05	0.36	**0.50**	−0.03	0.02	0.38
12. My parents tell me that my poor performance would damage the family’s honor	**0.41**	0.17	0.06	0.42	**0.40**	0.30	0.00	0.50
13. If I make them unhappy, my parents stop talking to me until I please them again	0.05	**0.68**	−0.16	0.41	0.02	**0.67**	−0.08	0.43
14. My parents often interrupt me	−0.26	**0.67**	0.20	0.44	−0.11	**0.55**	0.24	0.44
15.My parents change the subject whenever I have something to say	0.08	**0.60**	−0.09	0.43	0.23	**0.46**	−0.10	0.41
16. My parents scold me when they are not satisfied with me	−0.13	**0.59**	0.29	0.51	−0.04	**0.55**	0.26	0.54
17. My parents are less friendly with me if I do not see things their way	0.17	**0.59**	−0.02	0.55	0.18	**0.66**	−0.08	0.59
18. I feel like my parents interfere in everything I do	−0.06	**0.59**	0.18	0.52	0.12	**0.47**	0.18	0.56
19. No matter what I think or do, my parents always give me negative comments	0.04	**0.57**	0.14	0.51	−0.18	**0.83**	0.10	0.60
20. My parents avoid looking at me when I have disappointed them	0.32	**0.56**	−0.23	0.49	0.23	**0.71**	−0.24	0.56
21. My parents act cold and unfriendly if I do something they do not like	0.24	**0.56**	−0.01	0.55	0.08	**0.63**	0.03	0.55
22. My parents think my thoughts are naive	0.00	**0.53**	0.03	0.32	−0.11	**0.58**	0.14	0.37
23. My parents never praise me	−0.12	**0.50**	0.06	0.34	−0.14	**0.67**	−0.01	0.34
24. My parents insist that I do things their way	0.28	**0.46**	0.03	0.54	0.19	**0.44**	0.13	0.54
25. My parents often compare me with others (e.g., themselves when they were young or children of my age)	−0.06	−0.04	**0.92**	0.69	0.00	−0.11	**0.92**	0.68
26. My parents like to compare me to other children that they approve of when I act against their wishes	0.05	−0.10	**0.91**	0.74	0.01	0.01	**0.86**	0.75
27. My parents compare me with children who are better than I am at certain things	0.01	0.04	**0.73**	0.59	−0.04	0.06	**0.75**	0.59
28. My parents ask me why I cannot be as good as other children (e.g., children of our relatives/their friends or my classmates)	0.12	0.03	**0.69**	0.67	0.14	0.01	**0.70**	0.68
29. My parents tell me that I am not as good as other children when I fall short of their expectations	0.13	0.14	**0.60**	0.72	0.07	0.26	**0.50**	0.64
30. When I misbehave, my parents tell me I am not as good as other children	0.17	0.14	**0.57**	0.69	0.14	0.20	**0.50**	0.67
Variance explained (%)	40.38	5.92	5.63		6.07	42.73	5.13	

RI, relational induction; HPC, harsh psychological control; SCS, social comparison shame; Com., communalities. Factor loadings greater than 0.40 are in bold.

The three factors explained a total of 51.92 and 53.93% of the variance in maternal and paternal subscales, respectively. Based on the item content, the three factors were labeled as “relational induction” which consisted of 12 items on guilt induction and shared shame, “harsh psychological control” which included 12 items on invalidation, constraining verbal expression, personal attack, love withdrawal, and domineering control, and “social comparison shame” which included six items on negative comparisons to others. Although most of the items originally on domineering control and love withdrawal loaded on the factor of “harsh psychological control,” there were two exceptions. Specifically, one item (i.e., My parents tell me that what they want me to do is the best for me so I need to follow their demands) originally assigned to domineering control, and another item (i.e., My parents say that they will love me more if I perform better) originally assigned to love withdrawal loaded on the factor of “relational induction.” The findings suggest that these two items also imply the interrelationship between parents and children.

### 3.2. CFA and invariance tests

As shown in Table 2, the three-dimensional structure resultant from EFA fit the data adequately (CFI = 0.91, TLI = 0.91, RMSEA = 0.06, SRMR = 0.04, for both maternal and paternal subscales). In comparison, the unidimensional model for both subscales showed inadequate model fitness with CFI and TLI values ranging between 0.82 and 0.84. Furthermore, the BIC values of the three-factor structure were much lower than that of the unidimensional model (maternal: ΔBIC = 519.30; paternal: ΔBIC = 620.98). In addition, the average factor loadings for “relational induction” (maternal = 0.73; paternal = 0.74), “harsh psychological control” (maternal = 0.75; paternal = 0.77), and “social comparison shame” (maternal = 0.81; paternal = 0.82) were above 0.70. As a result, the three-factor structure was retained and used for further invariance tests.

**Table 2:** Model comparisons in confirmatory factor analyses (subsample B; *n* = 482).

Models	*χ^2^*	*df*	BIC	CFI	TLI	RMSEA	RMSEA 90% CI	SRMR	ΔBIC
Maternal subscale									
A. Unidimensional structure	1619.11	403	39339.38	0.84	0.83	0.08	0.077, 0.086	0.05	
B. Three-factor structure resultant from EFA	1081.45	400	38820.08	0.91	0.91	0.06	0.057, 0.066	0.04	519.30
Paternal subscale									
A. Unidimensional structure	1704.87	403	38294.92	0.83	0.82	0.08	0.080, 0.089	0.06	
B. Three-factor structure resultant from EFA	1065.54	400	37673.94	0.91	0.91	0.06	0.056, 0.065	0.04	620.98

df, degree of freedom; BIC, Bayesian information criterion; CFI, comparative fit index; TLI, Tucker-Lewis index; RMSEA, root mean square error of approximation; SRMR, standardized root mean square residual; CI, confidence interval; ΔBIC, change in BIC.

According to the results of invariance tests shown in Table 3, the three-factor structure demonstrated adequate fitness to data among male and female adolescents for both maternal and paternal subscales. In invariance tests across gender groups, differences in CFI and RMSEA between all pairs of nested models in sequential invariance tests were below 0.01 and 0.015 respectively, implying scalar (or strong) invariance (i.e., equal factor loadings and item intercepts) across gender groups regarding the three-factor structure of maternal and paternal CPPCS. The scalar invariance model showed acceptable fitness indices (maternal: *χ^2^*_(854)_ = 2217.48, CFI = 0.90, TLI = 0.90, RMSEA = 0.06, SRMR = 0.05; paternal: *χ^2^*_(854)_ = 2136.90, CFI = 0.91, TLI = 0.91, RMSEA = 0.06, SRMR = 0.05). Similar findings were observed for invariance tests across grade in both maternal and paternal subscales (see Table 3). Thus, the invariance across gender and grade was established.

**Table 3:** Invariance tests across gender groups and grades for the three-factor structure (Whole sample, *n* = 963).

	*χ^2^*	*df*	CFI	TLI	SRMR	RMSEA	RMSEA 90% CI	Compare	Δ*χ^2^*	ΔCFI	Δ*df*	ΔRMSEA
Maternal subscale												
Full sample (*n* = 963)	1552.58	400	0.92	0.91	0.04	0.06	0.053, 0.059					
Males (*n* = 460)	972.39	400	0.91	0.90	0.05	0.06	0.054, 0.064					
Females (*n* = 503)	1147.69	400	0.90	0.90	0.05	0.06	0.060, 0.068					
A. Configural	2137.18	800	0.90	0.90	0.05	0.06	0.059, 0.065					
B. Metric	2170.10	827	0.90	0.90	0.05	0.06	0.058, 0.064	B vs. A	32.92	0.000	27	−0.001
C. Scalar	2217.48	854	0.90	0.90	0.05	0.06	0.058, 0.064	C vs. B	47.38	−0.002	27	0.000
Grade 7 (*n* = 560)	1117.90	400	0.91	0.90	0.04	0.06	0.054, 0.062					
Grade 8 (*n* = 403)	1004.35	400	0.90	0.90	0.05	0.06	0.059, 0.069					
D. Configural	2139.74	800	0.91	0.90	0.05	0.06	0.058, 0.064					
E. Metric	2179.27	827	0.91	0.90	0.05	0.06	0.057, 0.063	E vs. D	39.53	−0.001	27	−0.001
F. Scalar	2217.74	854	0.91	0.90	0.05	0.06	0.056, 0.062	F vs. E	38.47	0.000	27	−0.001
Paternal subscale												
Full sample (*n* = 963)	1522.20	400	0.92	0.92	0.04	0.06	0.053, 0.059					
Males (*n* = 460)	1013.19	400	0.90	0.90	0.05	0.06	0.057, 0.066					
Females (*n* = 503)	1055.63	400	0.92	0.91	0.04	0.06	0.055, 0.064					
A. Configural	2068.83	800	0.91	0.90	0.05	0.06	0.057, 0.064					
B. Metric	2094.06	827	0.91	0.91	0.05	0.06	0.056, 0.063	B vs. A	25.23	0.001	27	−0.001
C. Scalar	2136.90	854	0.91	0.91	0.05	0.06	0.056, 0.062	C vs. B	42.84	−0.002	27	0.000
Grade 7 (*n* = 560)	1078.67	400	0.92	0.91	0.04	0.06	0.052, 0.060					
Grade 8 (*n* = 403)	1084.18	400	0.90	0.89	0.05	0.07	0.063, 0.073					
D. Configural	2162.85	800	0.91	0.90	0.05	0.06	0.058, 0.064					
E. Metric	2219.46	827	0.91	0.90	0.05	0.06	0.058, 0.064	E vs. D	56.61	−0.002	27	0.000
F. Scalar	2257.22	854	0.91	0.90	0.05	0.06	0.057, 0.063	F vs. E	37.76	0.000	27	−0.001

df, degree of freedom; CFI, comparative fit index; TLI, Tucker-Lewis index; RMSEA, root mean square error of approximation; SRMR, standardized root mean square residual; CI, confidence interval; Δχ^2^, change in χ^2^; ΔCFI, change in CFI; Δdf, change in df; ΔRMSEA, change in RMSEA.

### 3.3. Validity, reliability, and predictions

Table 4 summarizes the psychometric properties of each dimension in the CPPCS, correlations among them in maternal and paternal subscales, and the correlations between subscales and other parental factors. The results suggested that all dimensions possessed good internal consistency characterized by moderate mean inter-item correlation (0.41–0.65) and high Cronbach’s α, composite reliability (CR), and McDonald’s ω values (0.89–0.92). In addition, the AVEs of three dimensions were above 0.50 and the three dimensions were highly correlated with each other (*r*s = 0.70–0.73, *p*s < 0.001), supporting the scale’s convergent validity.

**Table 4:** Reliability of the Chinese Parental Psychological Control Scale and correlations among subscales and other parental factors (Whole sample, *n* = 963).

Subscales	No. of items	Reliability/AVE				Inter-correlations			Correlations with criteria			
		Maternal		Paternal		RI	HPC	SCS	Maternal		Paternal	
		MIC	α/CR/ω/AVE	MIC	α/CR/ ω/AVE				REJ	WAR	REJ	WAR
RI	12	0.41	0.89/0.89/0.90/0.54	0.42	0.90/0.89/0.89/0.55	1.00	0.73^***^	0.71^***^	0.69^***^	−0.13^***^	0.70^***^	−0.12^**^
HPC	12	0.43	0.90/0.90/0.91/0.57	0.45	0.91/0.91/0.90/0.60	0.73^***^	1.00	0.70^***^	0.86^***^	−0.37^***^	0.85^***^	−0.33^***^
SCS	6	0.66	0.92/0.92/0.92/0.65	0.66	0.92/0.92/0.92/0.65	0.72^***^	0.70^***^	1.00	0.68^***^	−0.24^***^	0.68^***^	−0.27^***^

RI, relational induction; HPC, harsh psychological control; SCS, social comparison shame; CR, composite reliability; AVE, average variance extracted; MIC, mean inter-item correlation; REJ, parental rejection; WAR, parental warmth. Inter-correlations of maternal and paternal subscales are shown below and above the diagonal, respectively. Maternal and paternal psychological control subscales were correlated to respective maternal and paternal rejection or warmth, respectively. ^**^p < 0.01, ^***^p < 0.001.

As shown in Table 4, maternal/paternal psychological control dimensions were positively correlated with maternal/paternal rejection. For both subscales, the harsh psychological dimension showed the highest correlation (maternal: *r* = 0.86, *p* < 0.001; paternal: *r* = 0.85, *p* < 0.001) with rejection than the other two dimensions. Meanwhile, the three dimensions of both maternal and paternal subscales were negatively correlated with maternal or paternal warmth, respectively. The harsh psychological control dimension also displayed the strongest negative correlations (maternal: *r* = −0.37, *p* < 0.001; paternal: *r* = −0.33, *p* < 0.001) while the relational induction dimension showed the weakest negative correlations (maternal: *r* = −0.13, *p* < 0.001; paternal: *r* = −0.12, *p* < 0.01). In general, these findings supported the concurrent validity of the CPPCS.

Moreover, differentiated predictions on adolescents’ developmental outcomes were observed for individual dimensions in the CPPCS (see Table 5). Specifically, for self-esteem and life satisfaction, the dimension of harsh psychological control and social comparison shame showed significant negative predictions while relational induction dimension demonstrated positive predictions. Furthermore, harsh psychological control was a significant positive predictor of adolescents’ depression while the other two dimensions were not. The findings supported the notion that relational induction is less detrimental than the other two dimensions among Chinese adolescents.

**Table 5:** Predictions of individual dimensions of psychological control on adolescents’ developmental outcomes (Whole sample, *n* = 963).

Coefficients [*β* (SE)]	Relational induction	Harsh psychological control	Social comparison shame
Self-esteem	0.26 (0.09)^**^ / 0.24 (0.09)^**^	−0.21 (0.08)^*^ / −0.21 (0.08)^*^	−0.20 (0.08)^*^/ −0.17 (0.07)^*^
Life satisfaction	0.24 (0.09)^**^ / 0.28 (0.09)^**^	−0.32 (0.08)^***^ / −0.38 (0.08)^***^	−0.21 (0.07)^**^ / −0.17 (0.07)^*^
Depression	−0.11 (0.09) / −0.12 (0.09)	0.43 (0.08)^***^ / 0.44 (0.08)^***^	0.04 (0.07) / 0.01 (0.07)

Maternal predictions are before the slash and paternal predictions are after the slash. ^*^p < 0.05, ^**^p < 0.01, ^***^p < 0.001.

## 4. Discussion

The present study reported findings of a validation study on the Chinese Parental Psychological Control Scale (CPPCS), which was developed to measure parents’ psychological control from all essential dimensions. The results indicated that the CPPCS has favorable psychometric properties, including good reliability and validity. Exploratory and confirmatory factor analyses supported the three-dimension structure of the CPPCS for both paternal and maternal scales, with the factor structure invariant across adolescent gender groups and all dimensions showing adequate internal consistency. Findings also supported the convergent validity of the CPPCS. Dimensions in paternal and maternal subscales were significantly correlated with each other and with parental warmth (positively) and rejection (negatively). The psychological control dimensions also showed divergent predictions on adolescents’ developmental outcomes, including well-being and ill-being measures. These findings suggested that the CPPCS is a useful tool for a differentiated approach to parental psychological control in Chinese contexts, especially for its full coverage of different controlling tactics and consideration of individual dimensions’ different functions.

In the present study, EFA and CFA yielded three interrelated but psychometrically distinct dimensions in the CPPCS, including “relational induction,” “harsh psychological control,” and “social comparison shame.” Although the three dimensions were closely correlated with each other, the unidimensional structure was not acceptable. The findings provide empirical support for the multidimensionality of parental psychological control, which is consistent with some previous conclusions (Yu et al., 2015; Sze, 2016). In addition, the “harsh psychological control” dimension seemed to have stronger positive and negative correlations with parental rejection and warmth, respectively, than the other two dimensions. This observation supports the thesis that different aspects of parents’ psychological control may not necessarily have the same meaning and function in Chinese contexts. In general, the present findings reiterate the need to distinguish between dimensions of psychological control rather than treating it as a global index or a unidimensional construct.

The resultant factor structure of the CPPCS echoes Fung and Lau’s (2012) proposition that relational induction behaviors are conceptually different from those more hostile ones (i.e., the “harsh psychological control” dimension). Apart from the above-mentioned different correlations between these two dimensions and the other two parental factors (rejection and warmth), “harsh psychological control” dimension also showed stronger negative predictions on adolescents’ developmental outcomes, while the “relational induction” dimension did not significantly predict depression and even positively predicted self-esteem and life satisfaction among adolescents. The preliminary findings on the differentiated functions support the notion that hostile forms of psychological control are universally detrimental for child development while relational induction in terms of guilt induction and shared shame may be more benign in Chinese contexts (Chen et al., 2016). Chinese parents are likely to use relational induction to draw children’s attention to parental sacrifices and the influence of their misbehavior on parental or familial reputation and honor, helping them acquire empathy for their parents and attunement to others’ perceptions, feelings, and thoughts (Fung and Lau, 2012; Yu et al., 2015). In a highly collectivist social milieu, this form of parenting is a strategic way to achieve socialization goals, which are likely to be culturally acceptable and thus less intrusive (Yu et al., 2019; Fang et al., 2022).

However, the present “relational induction” included tactics of guilt induction and shared shame, but not love withdrawal, which is different from Fung and Lau’s (2012) classification. Instead, love withdrawal loaded on the “harsh psychological control” dimension together with other hostile tactics. Such differences reinforce the need to explore factor structure using EFA prior to performing CFA, which has been grossly overlooked in previous studies (e.g., Fung and Lau, 2012; Yu et al., 2015; Fang et al., 2022). Moreover, the results support our expectation that love withdrawal is theoretically more aversive than guilt induction and shared shame. Prior research has suggested that love withdrawal, in comparison to guilt induction and shaming, was less likely to be perceived by children and adolescents as well-intended (Rohner et al., 2005; Cheah et al., 2019). Thus, some inconsistent findings in previous research may be partially due to the problematic assumption of similar conceptual meanings and functions of love withdrawal and guilt induction (Wang et al., 2007; Li et al., 2016; He et al., 2019; Gao et al., 2022).

The present findings also provide empirical evidence for the notion that “social comparison shame” is a unique dimension of parental psychological control (Fang et al., 2022), despite that most previous research did not include this type of tactics (Barber et al., 2005; Shek, 2006; Yu et al., 2021) or mixed it with shared shame (Yu et al., 2015; Sze, 2016). Our findings imply that social comparison shame is conceptually different from shared shame. It draws children’s attention to their inferiority and shortcomings compared to others, such as siblings or peers. Despite the fact that upward comparison may communicate parental expectations relative to well-mannered or high-achieving role models, negative labeling accompanied by potentially excessive demands makes children more likely to experience this type of parenting as disrespect, denigration, personal attack, or rejection (Smetana et al., 2021). This may be the reason that social comparison shame also negatively predicted adolescents’ self-esteem and life satisfaction in the present study.

Overall, the validated CPPCS and related findings in the present study provide additional empirical evidence to support the multifaceted nature of parental psychological control and culture-specific conceptualizations of its subtypes. This theoretical contribution also highlights the need to differentiate unique functions of individual dimensions of parental psychological control on Chinese adolescents, which has been ignored in previous studies and may be a promising way to resolve the existing inconsistent findings. Most importantly, the validated scale serves as a useful instrument that can be adopted in future studies involving Chinese adolescents to understand Chinese parents’ usage of different forms of psychological control and its correlates, impacts of individual dimensions on children’s and adolescents’ development, and the underlying mechanisms (e.g., moderating and mediating effects).

The present study has several limitations. First, participants were from only two grades in four secondary schools based on a convenience sampling strategy, which limits the application of the CPPCS and generalization of the findings in other Chinese samples. Future studies will benefit from using a more representative adolescent sample recruited by probability sampling methods such as stratified sampling. Second, when we formed the initial item pool, we only consulted 19 adolescents. It is also possible that the interpretation of parental psychological control varies across samples with different social background (e.g., mainland China versus Hong Kong; rural versus urban), age, and family structure (Cheah et al., 2019; Fang et al., 2022). Given adolescents’ perception and interpretation are essential in constructing correct and comprehensive items on parental psychological control (Barber et al., 2012), future studies may further validate the CPPCS by interviewing more adolescents with different backgrounds. In addition, the present study only validated adolescent-reporting version of the scale, future study can further construct and validate a parent-reporting version. In this way, data can be collected through not only adolescents’ self-reporting but also parental reporting.

Third, the present study only tested multi-group factorial invariance with cross-sectional data. Given the increasing emphasis on assessment tool’s longitudinal invariance, which enables a given scale to assess the same construct with the same structure across time (Widaman et al., 2010; Millsap and Cham, 2012), there is a need to test the longitudinal invariance of the CPPCS in future studies. Fourth, the present study only used two additional parental factors (rejection and warmth) and three adolescents’ outcome measures (self-esteem, life satisfaction, and depression) to test concurrent and predictive validities, respectively. Future studies need to adopt more criteria measures, including parental measures that are proximal to psychological control such as overparenting, harsh parenting, and autonomy support as well as adolescents’ outcome indicators (e.g., anxiety and academic achievement). In particular, there is a need to create a short version of the current 60-item scale (30 for paternal and maternal subscales, respectively) to reduce participants’ burden and increase the applicability of the CPPCS. Thus, in validating the abbreviated version, more criteria measures should be employed.

## 5. Conclusion

Despite these limitations, the validated CPPCS is a valuable addition to the existing conceptualization and assessment of parental psychological control among Chinese samples. The scale can serve as a useful tool for differentiated research on culture-specific meanings and functions of parental psychological control. Future studies will also benefit from developing a shortened form of the scale that retains the strong psychometric properties of the full-length scale but will be more concise.

## Data availability statement

The raw data supporting the conclusions of this article will be made available by the authors, without undue reservation.

## Ethics statement

The studies involving human participants were reviewed and approved by the Institutional Review Board at the Hong Kong Polytechnic University. Written informed consent to participate in this study was provided by the participants’ legal guardian/next of kin.

## Author contributions

XZ designed the research and contributed to all the steps of the work. DD contributed to the data collection and editing the manuscript. YP helped revise the manuscript. All authors contributed to the article and approved the submitted version.

## Funding

This research is financially supported by the Departmental General Research Fund (Project ID: P0041402) and the Undergraduate Research and Innovation Scheme (Project ID: P0043653) to XZ in the Department of Applied Social Sciences, the Hong Kong Polytechnic University.

## Conflict of interest

The authors declare that the research was conducted in the absence of any commercial or financial relationships that could be construed as a potential conflict of interest.

## Publisher’s note

All claims expressed in this article are solely those of the authors and do not necessarily represent those of their affiliated organizations, or those of the publisher, the editors and the reviewers. Any product that may be evaluated in this article, or claim that may be made by its manufacturer, is not guaranteed or endorsed by the publisher.
